# Mesmerism

**Published:** 1845-04

**Authors:** 


### MESMERISM.

Miss Martineau's case, copied from the London Lancet in our last number, doubtless attracted the attention of our readers. A case has lately occurred in New York which is exciting a good deal of speculation. It was a surgical operation performed on a young lady, by a young Frenchman, in the "Mesmeric Sleep," and which was first published, we believe, by Mr. N. P. Willis, in the National Intelligencer. Dr. Doane has subsequently reported the case in the Boston Medical and Surgical Journal. The operation consisted in removing a tumor as large as a "pullet's egg" from the neck, behind the jaw, which was done in two and a half minutes. The patient is said to have "slept profoundly" during the time, and to have manifested no sign of suffering. But the sleep is questioned by some, and the operation is thought to be "trivial to furnish a satisfactory test or proof of insensibility." We copy from the New York Journal of Medicine, the editor of which promises a full detail of the case in his next number. Meantime, he publishes an interesting letter from Professor Paine, giving the particulars of a most painful operation by Professor Mott—the exsection of the inferior maxillary bone—which the subject bore for an hour and a half, without manifesting the "slightest evidence of pain, or of impatience, or of fatigue, by winking, groaning or sighing." This case Professor P. relates as a rebuke to the mesmerite for "his love of the marvellous, or his love of mischief, or his yet more culpable designs on human credulity." Mesmerism he considers "one of the greatest nuisances that has yet infected the moral and reflecting part of the community." Y.

